# Impaired Osteoblast Function in *GPRC6A* Null Mice

**DOI:** 10.1359/jbmr.091037

**Published:** 2009-10-26

**Authors:** Min Pi, Lishu Zhang, Shu-Feng Lei, Min-Zhao Huang, Wenyu Zhu, Jianghong Zhang, Hui Shen, Hong-Wen Deng, L Darryl Quarles

**Affiliations:** 1 Department of Medicine, University of Tennessee Health Science Center Memphis, TN, USA; 2 College of Life Sciences and Bioengineering, Beijing Jiaotong University Beijing, People's Republic of China; 3 School of Medicine, University of Missouri–Kansas City Kansas City, MO, USA; 4 Vanderbilt University, Center for Bone Biology, Clinical Pharmacology, Division/Medicine Nashville, TN, USA

**Keywords:** GPRC6A, G protein–coupled receptor (GPCR), osteoblast, bone mineral density, gene polymorphisms

## Abstract

GPRC6A is a widely expressed orphan G protein–coupled receptor that senses extracellular amino acids, osteocalcin, and divalent cations in vitro. *GPRC6A* null (*GPRC6A*^−/−^) mice exhibit multiple metabolic abnormalities including osteopenia. To investigate whether the osseous abnormalities are a direct function of GPRC6A in osteoblasts, we examined the function of primary osteoblasts and bone marrow stromal cell cultures (BMSCs) in *GPRC6A*^−/−^ mice. We confirmed that *GPRC6A*^−/−^ mice exhibited a decrease in bone mineral density (BMD) associated with reduced expression of *osteocalcin*, *ALP*, *osteoprotegerin*, and *Runx*2-II transcripts in bone. Osteoblasts and BMSCs derived from *GPRC6A*^−/−^ mice exhibited an attenuated response to extracellular calcium-stimulated extracellular signal-related kinase (ERK) activation, diminished alkaline phosphatase (ALP) expression, and impaired mineralization ex vivo. In addition, siRNA-mediated knockdown of *GPRC6A* in MC3T3 osteoblasts also resulted in a reduction in extracellular calcium-stimulated ERK activity. To explore the potential relevance of GPRC6A function in humans, we looked for an association between *GPRC6A* gene polymorphisms and BMD in a sample of 1000 unrelated American Caucasians. We found that *GPRC6A* gene polymorphisms were significantly associated with human spine BMD. These data indicate that GRPC6A directly participates in the regulation of osteoblast-mediated bone mineralization and may mediate the anabolic effects of extracellular amino acids, osteocalcin, and divalent cations in bone. © 2010 American Society for Bone and Mineral Research.

## Introduction

GPRC6A is a recently identified member of family C of G protein–coupled receptors (GPCRs) that senses extracellular cations, osteocalcin, and amino acids.(1–4) Transcripts for *GPRC6A* are expressed in many tissues and organs, including lung, liver, spleen, heart, kidney, blood vessels, skeletal muscle, testis, brain, and bone.(1–5) Consistent with its broad expression, ablation of *GPRC6A* is associated with multiple abnormalities, including glucose intolerance, hepatic steatosis, abnormal steroid biogenesis, and osteopenia, suggesting that GPRC6A may directly or indirectly regulate anabolic responses in multiple organs.(6)

Bone has been proposed to be a special tissue compartment where the combination of calcium, osteocalcin, and amino acids might constitute important extracellular signals regulating bone formation.(4) While osteoblast-mediated bone formation is coupled to osteoclast-mediated bone resorption through the production by osteoblastic stromal cells of osteoprotegerin (OPG) and receptor activator of NF-κB ligand (RANKL),(7) there is also emerging evidence for reverse coupling by factors released from osteoclast-mediated degradation of mineralized bone matrix, such as amino acids and calcium, that act on osteoblasts to fill in the resorptive cavity.(8) Indeed, several mouse models with a primary increase in osteoclast-mediated bone resorption, including *OPG* and *calcitonin* null mice,(9,10) have a secondary increase in osteoblast-mediated bone formation. Conversely, osteopetrotic disorders caused by a primary decrease in bone resorption are often accompanied by decreased bone formation, possibly through the loss of signals from osteoclasts.(8) In addition, high ambient Ca^2+^ concentrations (in the range of 8 to 40 mM) and amino acids are present at sites of bone resorption,(11) and a positive correlation exists between lumbar and femoral bone mass and the intake of protein and calcium.(12) Since dietary protein-derived chemical signals may be derived from their metabolism into free amino acids,(13) circulating levels of amino acids and calcium also may modulate signaling pathways in bone. Finally, both osteoblasts and osteoclasts respond to extracellular calcium in vitro through a putative extracellular amino acid and calcium-sensing GPCR.(11,14,15)

GPRC6A is expressed in osteoblasts,(4,16) but its function in bone is not clear. Preliminary characterization of the skeleton of *GPRC6A*^−/−^ mice indicates that loss of this receptor is associated with decreased bone mineral density (BMD) and impaired mineralization of bone,(6) but the presence of other abnormalities confounded the ability to ascertain the direct and indirect effects of GPRC6A on skeletal function. To determine if GPRC6A is a potential candidate for the purported extracellular calcium-sensing receptor in osteoblasts, we performed a more detailed assessment of the skeletal phenotype of *GPRC6A*^−/−^ mice, examined the function of primary osteoblasts and bone marrow stromal cells derived from these mutant mice ex vivo, and evaluated whether polymorphisms in *GPRC6A* are associated with skeletal abnormalities in humans by a gene association analysis.

## Materials and Methods

### *GPRC6A* knockout mice

The *GPRC6A*-deficient mouse model was created by replacing exon 2 of the *GPRC6A* gene with the hygromycin resistance gene, as described previously.(6) Mice were maintained and used in accordance with recommendations of theNational Research Council's (1985) *Guide for the Care and Use of Laboratory Animals* (DHHS Publication NIH 86-23, Institute on Laboratory Animal Resources, Rockville, MD) and following guidelines established by the University of Kansas Medical Center Institutional Animal Care and Use Committee.

### RT-PCR and real-time RT-PCR

Reverse-transcriptase polymerase chain reaction (RT-PCR) was performed using two-step RNA PCR (Perkin-Elmer, Waltham, MA, USA). In separate reactions, 2.0 µg of DNase-treated total RNA was reverse transcribed into cDNA with the respective reverse primers specified below and Moloney murine leukemia virus reverse transcriptase (Life Technologies, Inc., Rockville, MD, USA). Reactions were carried out at 42°C for 60 minutes followed by 94°C for 5 minutes and 5°C for 5 minutes. The products of first-strand cDNA synthesis were directly amplified by PCR using AmpliTaq DNA polymerase (Perkin-Elmer). The primer sets used to amplify various gene transcripts with intron spanning are as follows: mGPRC6A.189F: CGGGAT CCAGACGACCACAAATCCAG and mGPRC6A.539R: CCAAGCTTGATTCATAACTCACCTGTGGC; mALP.905F: AACCCAGACACAAGCATTCC and mALP.1458R: CTGGGCCTGGTAGTTGTTGT, G3PDH.F143: GACCCCTTCATTGACCTCAACTACA; and G3PDH.R1050: GGTCTTACTCCTTGGAGGCCATGT for control RNA loading.

For quantitative real-time RT-PCR assessment of bone marker expression, we isolated and reverse transcribed 2.0 µg of total RNA from the long bones of 8-week-old mice as described previously.(17) PCR reactions contained 100 ng of template (cDNA or RNA), 300 nM each of forward and reverse primer, and 1× iQ SYBR Green Supermix (Bio-Rad, Hercules, CA, USA) in 50 µL. Samples were amplified for 40 cycles in an iCycler iQ Real-Time PCR Detection System (Bio-Rad) with an initial melt at 95°C for 10 minutes, followed by 40 cycles of 95°C for 15 seconds and 60°C for 1 minute. PCR product accumulation was monitored at multiple points during each cycle by measuring the increase in fluorescence caused by the binding of SybrGreen I to dsDNA. The threshold cycle (*C*_*t*_) of tested-gene product from the indicated genotype was normalized to the *C*_*t*_ for cyclophilin A. Dissociation analysis was used to confirm the presence of a single transcript and lack of primer-dimer amplification in all PCR reactions.

### PIXImus bone densitometer analysis, X-ray, and bone histology

Bone mineral density (BMD) values of whole skeletons and femurs were assessed at 6, 8, 12, and 16 weeks of age using a PIXImus bone densitometer (Lunar Corp., Madison, WI, USA) as described previously.(18) Femurs were dissected free of muscle and X-rayed with a Faxitron model MX-20 specimen radiography system (Faxitron X-Ray Corp., Lincolnshire, IL, USA). In addition, length and width (measured at 50% of the femur length) were assessed in isolated femora. Skeletons of mice were prelabeled twice with calcein (Sigma C-0875, 30 µg/g of body weight; Sigmal Chemical Company, St. Louis, MO, USA) by intraperitoneal injection at 8 and 3 days prior to sacrifice. Tibiae and femora were removed from 8- and 16-week-old mice, fixed in 70% ethanol, prestained in Villanueva stain, and processed for methyl methacrylate embedding. Then 10-µm Villanueva-prestained sections were evaluated under fluorescent light, as reported previously by our laboratory.(18)

### Micro-computed tomographic (µCT) analysis

The distal femoral metaphyses were scanned using a µCT 40 device (Scanco Medical AG, Wayne, PA, USA); 167 slices of the metaphysis under the growth plate, constituting 1.0 mm in length, were selected. The 3D images were generated using the following values for a gauss filter (σ = 0.8, support 1) and a threshold of 275. A 3D image analysis was performed to determine bone volume (BV/TV), trabecular number (Tb.N), trabecular thickness (Tb.Th), and trabecular separation (Tb.Sp). Cortical bone was measured on the midshaft region of cortical bone in 50 slices of the diaphysis, constituting 0.3 mm in length. The mean cortical thickness (Ct.Th) was determined at eight different points on the cortical slice.

### Primary bone marrow stromal cells and osteoblastic cells culture

The femora and tibiae from 8-week-old wild-type and *GPRC6A*^−/−^ mice were dissected, the ends of the bones were cut, and marrow was flushed out with 2 mL of ice-cold α modified essential medium (α-MEM) containing 10% fetal bovine serum (FBS) by using a needle and syringe. A suspension of bone marrow cells was obtained by repeated aspiration of the cell preparation through a 22G needle, and nucleated cells were counted with a hemocytometer. Cells were seeded into 6-well plates at a density of 3 × 10^7^ cells/mL and cultured for 3 days in α-MEM supplemented with 10% FBS, 100 kU/L of sodium penicillin G, and 100 mg/L of streptomycin sulfate in a humidified incubator with 5% CO_2_ and 95% air at a temperature of 37°C. On day 3, all nonadherent cells were removed with the first medium change, and then the adherent cells [representing bone marrow–derived mesenchymal stem cells (BMSCs)] were grown for additional periods of up 3 days in the same medium. After overnight quiescence, the cells were stimulated for 5 minutes with calcium, NPS-R568, and arginine at the concentrations indicated.

We used modifications of a nonenzymatic method for obtaining the osteoblastic cell lines.(19) A fragment of the frontal and/or parietal bone from the single calvarium was aseptically removed from a 3- to 7-day-old mouse. Suture lines and endosteum were dissected away, and the bone fragment was placed in a culture dish. One or two metal strips were positioned on the endocranial surface and incubated for 3 to 4 days in DMEM nutrient mixture F-12 (Invitrogen, Carlsbad, CA, USA) containing 10% (v/v) FBS, 100 kU/L of sodium penicillin G, and 100 mg/L of streptomycin until the outgrowth of osteoblasts. The metal strips were removed, and the cells were allowed to grow until approximately 60% confluent. The cells were subcultured and propagated by incubation in α-MEM (Invitrogen) containing 10% FBS, 100 kU/L of sodium penicillin G, 100 mg/L of streptomycin, and 50 µg/mL ascorbic acid in a humidified atmosphere of 5% CO_2_ and 95% air at 37°C.

### Agonist stimulation and Western blotting

Agonist stimulation was performed in quiescent cells. Quiescence was achieved in subconfluent cultures by removing the medium and washing with Hank's balanced salt solution (Invitrogen) to remove residual serum, followed by incubation for an additional 24 hours in serum-free medium. After agonist treatment at the specified concentrations and durations, cells were washed twice with ice-cold PBS and scraped into 250 µL of lysis buffer (25 mM HEPES, pH 7.2, 5 mM MgCl_2_, 5 mM EDTA, 1% Triton X-100, and 0.02 tablet/mL of protease inhibitor mixture). Equal amounts of lysates were subjected to 10% SDS-PAGE and transferred onto Immun-Blot PVDF (Bio-Rad). For phospho-ERK, the phospho-ERK1/2 levels were determined by immunoblotting using anti-phospho-ERK1/2 mitogen-activated protein kinase antibody (Cell Signaling Technology, Inc., Beverly, MA, USA). To confirm that variations in the amount of ERK did not contribute to stimulated ERK activity, we used an anti-ERK1/2 mitogen-activated protein kinase antibody (Cell Signaling Technology, Inc.) to measure ERK levels for control protein loading. The signals were quantified by densitometric scanning (FotoDyne, Hartland, WI, USA).

### Mineralization assay

The formation of in vitro mineralization nodules was determined by alizarin red S histochemical staining.(15) The 14-day cultured cells in the α-MEM containing 10% FBS, 100 kU/L of sodium penicillin G, 100 mg/L of streptomycin sulfate, 50 µg/mL of ascorbic acid, and 4 mM of arginine were fixed for 24 hours in a solution of 10% Formalin, methanol, and water (1:1:1.5); the fixative was removed; and the fixed cells and matrices were stained for 15 minutes with a 2% (w/v) solution of alizarin red S at pH 4.0. The stained samples were washed three times with water and then air-dried.

### Immunohistochemistry for osteocalcin (OC) and measurement of aklaline phosphatase (ALP) activity

Anti-osteocalcin antibody was obtained from Dr Larry Fisher (NIDCR, National Institutes of Health). After deparaffinization and rehydration, the bone sections were immersed in 3% hydrogen peroxide to quench endogenous peroxidase and further digested with 1 µg/mL trypsin for 30 minutes at 37°C. Sections then were blocked with 1% bovine serum albumin at room temperature for 2 hours. The primary antibodies were added to the sections and incubated overnight at 4°C. After washing, the sections were coated with biotinylated second antibody (Vector Laboratories, Burlingame, CA, USA) at a dilution of 1:200 and then incubated at room temperature for 60 minutes. The sections were washed again and incubated with the ABC reagent (Vector Laboratories, Burlingame, CA, USA) at room temperature for 60 minutes. The 3,3'-diaminobenzidine substrate was used to visualize immunoreaction sites. Sections were counterstained with hematoxylin and mounted on glass slides. Negative controls were obtained by substituting the primary antibody with normal IgG.

Alkaline phosphatase (ALP) activity was detected by an enzyme-substrate assay in frozen sections of bone. Briefly, fresh nondecalcified spines from 8-week-old wild-type and *GPRC6A* knockout mice were dissected and embedded with Optimum Cutting Temperature (OCT, Sakur Finetek, USA, Inc., Torrance, CA, USA) medium. ALP activity was detected in 12-µm-thick cryostat sections at alkaline pH by adding ALP substrate 4-nitro blue tetrazolium (NBT) and 5-bromo-4-chloro-3-indolyl phosphate (BCIP) directly on the slides according to the manufacturer's instructions (Roche Applied Science, Indianapolis, IN, USA). Endogenous ALP activity was visualized as a purple-blue color reaction with methyl green (nuclei) counterstain.

### In situ hybridization for *osteocalcin* mRNA expression

A mouse *osteocalcin* cDNA plasmid, *Eco*RI/*Pst* I subclone inserted into pSP65, was obtained from Dr Stephen Harris (University of Texas Health Science Center, San Antonio, TX, USA). A digoxigenin (DIG)–labeled cRNA probe was prepared by using the RNA Labeling Kit (Roche Applied Science). To make the antisense probe, a partial mouse *osteocalcin* cDNA (0.5 kb) was cleaved by *Hin*d III and labeled with Sp6 polymerase. Sections of the tibia from a 8-week-old mouse were dewaxed, rehydrated, fixed with 4% paraformaldehyde, treated with 2% glycine and proteinase K, acetylated using an acetic anhydride–TEA solution, and then hybridized with a DIG-labeled probe. After washing the sections twice with 50% formamide, 5% saline sodium citrate, and 5% SDS for 30 minutes at 70°C and once with 50% formamide and 2% saline sodium citrate for 30 minutes at 65°C, we incubated them with antibody to digoxigenin conjugated with alkaline phosphatase and then with nitro blue etrazolium/4-bromo-5-chloro indolylphosphate, which yields a purple-blue color. We also counterstained the sections with methyl green (nuclei). The hybridization signals were photographed with a Nikon E800 microscope (Nikon, Melville, NY, USA) and a MagnaFire camera (Optronics, Muskogee, OK, USA).

### Serum and urine biochemical measurements

Serum was collected using a retro-orbital bleeding technique. For urine samples collection, mice were placed in metabolic cages (Hatteras Instrument, Cary, NC, USA), and urine was collected for 24 hours. The urine volume was measured before storage at −70°C.

Serum and urinary calcium was measured by the colorimetric cresolphthalein-binding method, and phosphorus was measured by the phosphomolybdate–ascorbic acid method.(18) Serum tartrate-resistant acid phosphatase (TRACP) was assayed with the ELISA-based SBA Sciences mouseTRAP assay (Immutopics, Inc., San Clemente, CA, USA). Serum parathyroid hormone (PTH) and 1,25-dihydroxyvitamin D were measured with an Immunodiagnostic Systems device (Immunodiagnostic Systems, Ltd., Scottsdale, AZ, USA). Serum Fgf23 levels were measured using an FGF-23 ELISA kit (Kainos Laboratories, Inc., Tokyo, Japan) following the manufacturer's protocol. Creatinine was measured by the colorimetric alkaline picrate method (Sigma Kit 555). Urinary protein and deoxypridinolme (Dpd) were measured by Bio-Rad and Metra Biosystems (Hercules, San Diego, CA, USA) devices, respectively.

### siRNA transfection

Two siRNAs (siRNA.m1638: CCAACACAGCTGTTGCTAT and siRNA.m2553: GCAGAAGACTAACACCAAA) specific for marine *GPRC6A* (GenBank Accession Number NM_153071), identified using the “siRNA Target Finder” at the Web site (http://www.ambion.com/techlib/misc/siRNA_finder.html), were used as a template for synthesizing siRNA. Double-stranded siRNA was synthesized from DNA oligonucleotides using the *Silencer* siRNA construction kit (Ambion, Austin, TX, USA) according to manufacturer's instructions.

For transfection, MC3T3 cells were plated at a density of 1.0 × 10^4^ cells per well in a 6-well dish and grown overnight. Cells were transiently transfected with 25 nM of siRNA using 4 µL of Lipofectamine 2000 (Invitrogen) for 4 hours, after which growth medium was replaced. Then, 48 hours after transfection, cells were quiescence overnight and then stimulated by 10 mM of calcium for 5 minutes and lysed for Western blot analysis. Scrambled siRNA (Select Negative Control 1 siRNA, Ambion) was used as negative control.

### Study populations and genotyping

The 1000 unrelated American Caucasians of European origin studied were identified from our established and expanding genetic repertoire at UMKC. Signed informed-consent documents were obtained from all study participants before they entered the study. The population genetic research on *GPRC6A* was approved by the Institutional Review Board. The inclusion and exclusion criteria are the same as those described elsewhere.(20) The basic characteristics of the studied subjects are listed in Supplemental Table 1. Briefly, the sample includes 251 elderly men (age range at recruitment 52.9 to 87.9 years), 250 younger men (age range at recruitment 19.1 to 49.9 years), 250 postmenopausal women (age range at recruitment 54.3 to 80.6 years), and 249 premenopausal women (age range at recruitment 19.2 to 53.2 years). Area BMD (g/cm^2^) at the spine and hip region was measured by dual-energy X-ray absorptiometry (DXA) with Hologic QDR 4500W densitometers (Hologic, Inc., Bedford, MA, USA). The scanners were calibrated daily. The coefficient of variation (CV) values of the DXA measurements were about 1.98% and 1.87% for spine and hip BMD, respectively.

The nine single-nucleotide polymorphisms (SNPs) of interest in vicinity of or inside the *GPRC6A* gene were genotyped successfully with the Affymetrix GeneChip human mapping 500K array set (Affymetrix, Inc., Santa Clara, CA, USA) as part of the large-scale genotyping project. The genotyping was performed at the Vanderbilt Microarray Shared Resource at Vanderbilt University Medical Center (Nashville, TN, USA) using the standard protocol recommended by the manufacturer. The genotyping procedure is detailed elsewhere.(20) The average per-SNP call rate of these nine SNPs across the 1000 subjects is 99.4% (97.2% to 99.9%).

### Statistical analysis

We evaluated differences between groups by one-way analysis of variance. All values are expressed as means ± SEM. All computations were performed using the Statgraphic Statistical Graphics System (STSC, Inc., Rockville, MD, USA).

The Hardy-Weinberg equilibrium (HWE) test and linkage disequilibrium (LD) plotting of the interested SNPs were performed using the Haploview 4.1 (Broad Institute, Cambridge, MA) .(21) The default option “confidence intervals” in Haploview 4.1 was used to define LD blocks.(22) Before performing the marker-trait association analysis, parameters such as age, age^2^, sex, age/age^2^-by-sex interaction, height, and weight were tested for their associations with BMD at the spine and hip; then the significant (*p* ≤ .05) terms were included as covariates to adjust the raw BMD values for subsequent analyses. All marker-trait association analyses were performed using association tests implemented in HelixTree 5.3.1 (Golden Helix, Bozeman, MT, USA). To circumvent the problem of false-positive results incurred by multiple testing, the SNPSpD program developed by Nyholt was used to establish the experiment-wise significance threshold needed to keep the type I error rate less than 5%, considering the redundancy of information provided by SNPs that were in LD.(23)

## Results

### Skeletal imaging

By X-ray analysis, we observed no significantly gross developmental abnormalities in the skeleton (1*A*). Consistent with prior reports,(6) BMD as assessed by dual-energy X-ray absorptrometry (DEXA) was significantly lower in 16-week-old *GPRC6A*^−/−^ mice compared with wild-type mice (1*B*). We observed no difference in femur length (1*C, D*), but there was a significant reduction in femur width between *GPRC6A*^−/−^ and wild-type mice (1*C, E*). In addition, we found that BMD was reduced by µCT in both the metaphyseal area, which predominately consists of trabecular bone, and the midshaft region, which is composed of cortical bone (1*F*). These differences were not due to effects of a mixed genetic background because these animals were crossed onto C57BL/6 mice for more than six generations. There were no demonstrable changes, however, in bone structural parameters, including bone volume (BV/TV) and cortical thickness (Ct.Th), between *GPRC6A*^−/−^ and wild-type mice, as assessed by µCT, suggesting that the decreased BMD was not due to structural changes in bone architecture.

**Fig. 1:**
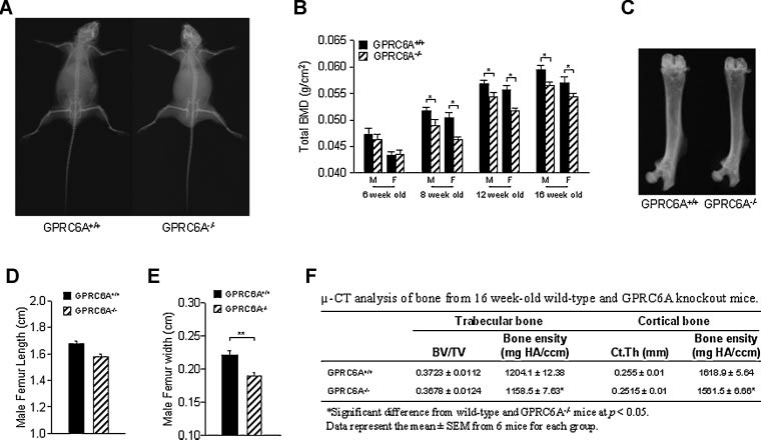
Characterization of the bone phenotype of *GPRC6A*^−/−^ mice. (*A*) X-ray appearance for *GPRC6A*^+/+^ and *GPRC6A*^−/−^ mice at 16 weeks of age. (*B*) Comparison of the total BMD analysis by PIXI_mus_ analysis in *GPRC6A*^+/+^ and *GPRC6A*^−/−^ mice at ages ranging from 6 to 16 weeks. (*C*) Abnormalities in femur bone of *GPRC6A*^−/−^ mice by X-ray. (*D*, *E*) Comparison of femur length (*D*) and femur width (*E*) in *GPRC6A*^+/+^ and *GPRC6A*^−/−^ mice at 16 weeks of age. (*F*) Comparison of the structure and BMD of the femur as assessed by µCT scanning in 16-week-old *GPRC6A^+/+^* and *GPRC6A*^−/−^ mice. Data represent the mean ± SEM from 6 to 10 mice in each group. ^*^Significant difference from *GPRC6A^+/+^* and *GPRC6A*^−/−^ mice at *p* < .05.

### Biomarkers/immunohistochemistry/gene expression

**Fig. 2:**
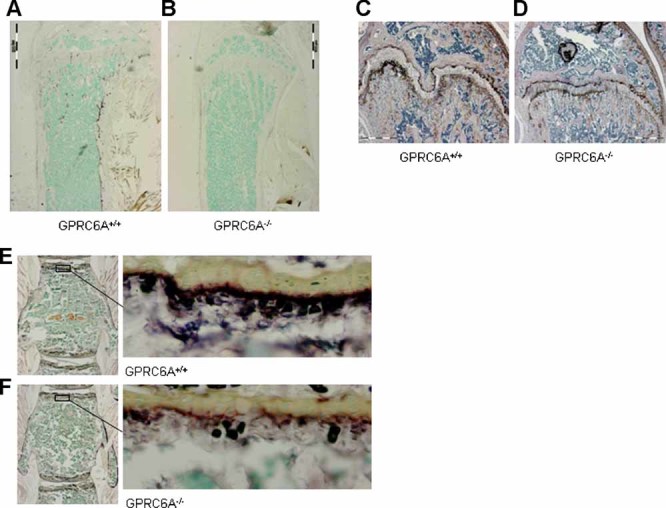
Decreased *osteocalcin* expression and *ALP* activaties in *GPRC6A*^−/−^ mice. (*A*, *B*) Comparison of *osteocalcin* message expression by in situ hybridization in the femora of *GPRC6A*^+/+^ (*A*) and *GPRC6A*^−/−^ mice (*B*). (*C*, *D*) Comparison of *osteocalcin* expression by immunohistochemistry in the femora of *GPRC6A*^+/+^ (*C*) and *GPRC6A*^−/−^ mice (*D*). (*E*, *F*) Comparison of *ALP* activity in the cryostat sections of fresh nondecalcified spines from *GPRC6A*^+/+^ (*E*) and *GPRC6A*^−/−^ mice (*F*). Right panels are enlarged view from left panels. The techniques for in situ, immunohistochemistry and measurement of alkaline phosphatase activity are described under “Materials and Methods.”

We found no evidence for *GPRC6A* effects on osteoclast function. In this regard, the osteoclastic marker tartrate-resistant acid phosphatase (*TRACP*) was not significantly different between wild-type and *GPRC6A*^−/−^ mice (Table 1), consistent with our prior findings that neither urinary Dpd/creatinine ratio nor serum TRACP levels were different between *GPR6CA*^−/−^ and *GPR6CA*^*+/+*^ mice.(6) To better understand the effect of GPRC6A on osteoblasts, we examined gene expression profiles of whole-bone samples from *GPRC6A*^−/−^ mice. Assessment of expression of osteoblast markers in bone from 16-week-old *GPRC6A*^−/−^ mice revealed reductions in *osteocalcin*, *ALP*, *osteoprotegerin*, and *Runx*2-II message levels compared with wild-type mice by real-time RT-PCR (Table 1), consistent with a reduction in mineral apposition rates, as reported previously.(6) We also confirmed by in situ hybridization and immohistochemical staining that *osteocalcin* expression (Fig. 2*A–D*) and *ALP* activity (2*E, F*) were reduced in bone from 16-week-old *GPRC6A*^−/−^ mice. The chondrocyte marker *Col*II and adipocyte markers *aP2* and *Lp1* were not significantly different between wild-type and *GPRC6A*^−/−^ mice (Table 1).

**Table 1:** Gene Expression Profile in Bone From *GPRC6A*^+/+^ and *GPRC6A*^−/−^ Mice

Gene	Accession number	*GPRC6A*^+*/*+^	*GPRC6A*^−/−^
*ALP*	NM_007431	0.493 ± 0.096	0.194 ± 0.0045^a^
*Osteocalcin*	NM_007541	1.101 ± 0.068	0.411 ± 0.1^a^
*Osteoprotegerin*	MMU94331	0.0755 ± 0.021	0.0159 ± 0.0045^a^
*Runx*2-II	NM_009820	0.156 ± 0.034	0.0563 ± 0.0061^a^
*Osterix*	AF184902	0.00187 ± 0. 00078	0. 00136 ± 0. 00047
*RANKL*	NM_011613	0.000987 ± 0.00011	0.00124 ± 0.00037
*TRACP*	NM_007388	0.793 ± 0.188	0.742 ± 0.12
*Col*II	NM_031163	0.457 ± 0.219	0.186 ± 0.056
*aP*2	NM_024406	1.229 ± 0.305	1.624 ± 0.342
*Lp*I	NM_008509	0.0803 ± 0.0149	0.118 ± 0.054

Data are mean ± SEM from 8-week-old mice. Values are expressed relative to the housekeeping gene *cyclophilin A*. *ALP* *=* alkaline phosphatase; *RANKL* *=* receptor activator for nuclear factor κB ligand; *TRACP* *=* tartrate-resistant acid phosphatase; *aP*2 = adipocyte fatty acid–binding protein 2; *Col*II = collagen type II; and *Lp*l = lipoprotein lipase.

a Denotes significant difference between *GPRC6A*^*+/+*^ and *GPRC6A*^−/−^ mice at *p* < .05.

### Impaired extracellular calcium response in BMSCs and calvarial-derived osteoblastic cells cultures from *GPRC6A*^−/−^ mice

**Fig. 3:**
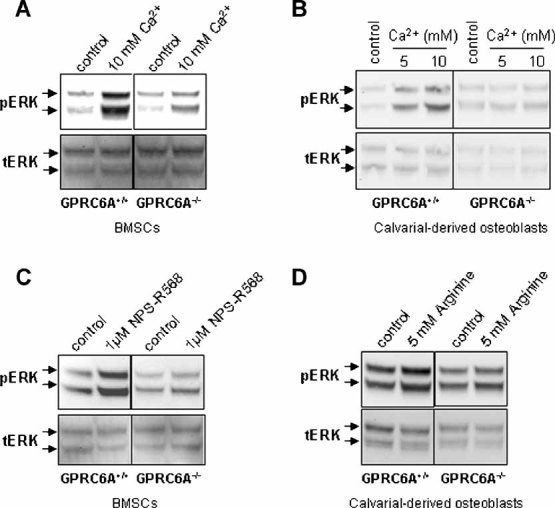
Decreased response to extracellular calcium, NPS-R568, and amino acid in BMSCs or osteoblasts from *GPRC6A*^−/−^ mice. (*A*, *B*) Bone marrow stromal cell cultures (BMSCs) and osteoblasts from *GPRC6A*^−/−^ mice displayed impaired responses to extracellular calcium-mediated ERK activation. (*C*) ERK activation in response to the calcimimetic NPS-R568 also was impaired in BMSCs derived from *GPRC6A*^−/−^ mice. (*D*) Osteoblasts derived from *GPRC6A*^−/−^ mice also showed impaired ERK activation in response to the amino acid arginine. ERK phosphosphorylation was assessed by Western blot analysis using an anti-phospho-ERK antibody. Data represent three to four independent experiments.

To explore whether the observed skeletal abnormalities are due to a primary osteoblastic abnormality owing to loss of *GPRC6A* in these cells, we evaluated the response to calcium, calcimimetic, and arginine of cultured of bone marrow stromal cell cultures (BMSCs) and calavarial osteoblasts obtained from wild-type and *GPRC6A*^−/−^ mice (Fig. 3). In both BMSCs and primary osteoblasts, we observed a reduced ability of extracellular calcium to stimulate ERK activity in *GPRC6A*^−/−^ mice compared with wild-type cells (approximately 50% reduction; 3*A, B*). In addition, a receptor allosteric modulator, the calcimimetic NPS-568, and the amino acid receptor ligand l-arginine both had an attenuated ERK activation in BMSCs and primary osteoblasts obtained from *GPRC6A*^−/−^ mice compared with wild-type cells (Figs. 3*C, D*).

To further evaluate osteoblast dysfunction in *GPRC6A*^−/−^ mice, we examined the impact of loss of *GPRC6A* on the capacity of cultured osteoblasts and BMSCs to undergo differentiation in vitro, as assessed by culture duration–dependent changes in alkaline phosphatase expression and activity. We found that wild-type osteoblasts and BMSC cultures increased alkaline phosphatase expression and activity during differentiation, but this increase was attenuated in *GPRC6A*^−/−^ cells (4*A, B*). These results are consistent with the gene expression data by real-time RT-PCR (Table 1) and immohistochemical staining (2*E, F*) in intact bone. Moreover, the addition of arginine increased mineralization in wild-type BMSC cultures but not in *GPRC6A*^−/−^ cells (4*C*). These data indicate that the lack of *GPRC6A* impairs the ability of osteoblasts to sense calcium and amino acids as well as their ability to undergo differentiation and form a mineralized extracellular matrix.

**Fig. 4:**
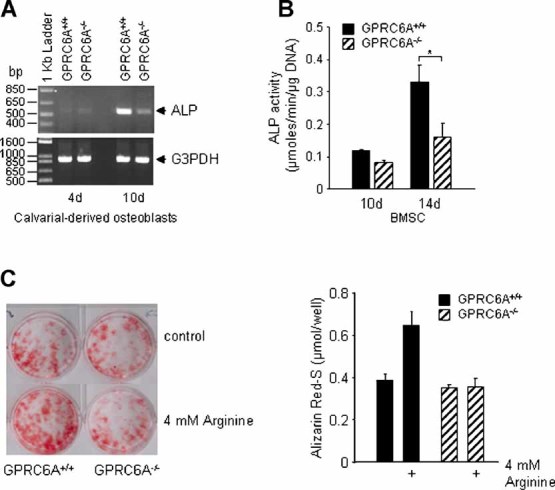
Characterization of temporal maturational sequence in osteoblasts or BMSCs from *GPRC6A^+/+^* and *GPRC6A*^−/−^ mice. (*A*) RT-PCR of alkaline *ALP* from 4- and 10-day cultured cells derived from 8-week-old *GPRC6A^+/+^* and *GPRC6A*^−/−^ calvaria. (*B*) *ALP* activity. The *GPRC6A*^−/−^ BMSCs had significantly lower *ALP* activity at days 10 and 14 of culture compared with age-matched wild-type mice. (*C*) Quantification of mineralization. Alizarin red S was extracted with 10% cetylpyridinium chloride and quantified as described under “Materials and Methods.” Data represent the mean ± SEM from three to four independent experiments.

### Response of osteoblastic cell line MC3T3 to extracellular calcium through GPRC6A

To confirm the role of GPRC6A as a relevant calcium- and amino acid–sensing receptor, we examined the MC3T3 clonal osteoblastic cell line, which is known to express a putative calcium-sensing receptor.(4,24) We examined the function of GPRC6A by siRNA-mediated knockdown in MC3T3 osteoblasts. We transfected MC3T3 cells with either *GPRC6A* siRNA.m2563 or siRNA.m1638. Mock-transfected cells and transfected cells with a random negative control siRNA plasmid were used as the control. Transfected MC3T3 cells *GPRC6A* siRNA.m2563 and siRNA.m1638 successfully downregulated the levels of mRNA expression of *GPRC6A* in MC3T3 cells compared with mock-transfected cells and negative-control siRNA-transfected cells by RT-PCR analysis (5*A*). In addition, the activation of phospho-ERK stimulated by extracellular calcium in MC3T3 cells was significantly decreased by both transfected *GPRC6A* siRNA.m2563 and siRNA.m1638 (reduced approximately 50% and 97%, respectively; 5*B*). These results suggest that endogenous GPRC6A accounts for the effects of extracellular calcium in stimulating ERK phosphorylation.

**Fig. 5:**
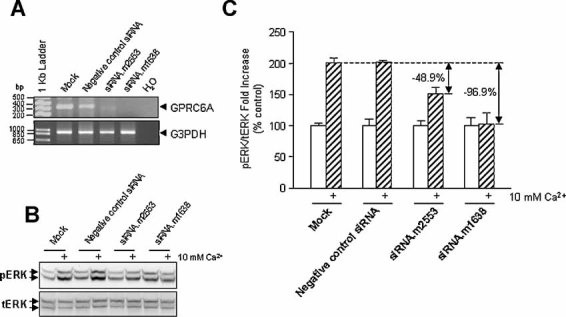
Knockdown of endogenous *GPRC6A* expression in cultured osteoblastic MC3T3 cells represses to response to extracellular calcium. (*A*) *GPRC6A* mRNA levels were analyzed by RT-PCR analysis in cultured osteoblastic MC3T3 cells transfected with indicated siRNAs for 48 hours. (*B*, *C*) siRNAs mediated knockdown of *GPRC6A-*inhibited extracellular calcium-stimulated phospho-ERK activation in cultured osteoblastic MC3T3 cells. Phospho-ERK levels were analyzed by Western blot analysis in cells transfected with indicated siRNA for 48 hours and then quiescent and stimulated with extracellular calcium as indicated under “Materials and Methods.” A minimum of three independent experiments was performed for each agonist and cell model. Panel *B* is a representative response from a single experiment, and panel *C* shows the mean ± SEM of the changes in band intensity from three separate experiments.

### Association of *GPRC6A* polymorphisms with spine and hip BMD

Next, we performed a gene association analysis to test whether *GPRC6A* gene polymorphisms are associated with the variation in human BMD.(25) Nine SNPs located from 21 kb downstream to 42 kb upstream of the *GPRC6A* gene were tested for their association with hip and spine BMD variation in the human subjects studied. All nine SNPs are in HWE (*p* > .01) with minor allele frequency (MAF) greater than 5%. The basic information on these SNPs is shown in Table 2.

**Table 2:** Associations of the Analyzed *GPRC6A* SNPs With Hip and Spine BMD^a^

No.	SNP name	Position (bp)	Role	Major/minor alleles	MAF^a^	*p* Value,^b^ hip BMD	*p* Value,^b^ spine BMD
1	rs686708	117198376	Downstream	A/G	0.453	**.0280**	**.0010**
2	rs571296	117199251	Downstream	A/G	0.344	**.0231**	**.0068**
3	rs587771	117212212	Downstream	G/T	0.205	.9168	**.0077**
4	rs6924002	117220916	CDS	A/T	0.307	.0713	**.0408**
5	rs17078383	117230654	Intron	C/T	0.058	.0909	**.0400**
6	rs6938235	117254639	Intron	G/T	0.330	**.0268**	**.0139**
7	rs17078405	117260764	Upstream	A/G	0.061	**.0400**	**.0244**
8	rs339319	117295428	Upstream	G/A	0.277	.6473	**.0483**
9	rs339321	117298883	Upstream	T/C	0.276	.6769	**.0400**

a MAF in our sample.

b *p* Values in bold indicate nominal significant associations (*p* ≤ .05). *p* Values in bold italic indicate significant associations after adjusting for multiple testing (*p* < .0074).

^a^ For the SNP rs686708, the adjusted BMD values for the three genotypes AA, AG, and GG in the samples studied were as follows: −0.015, 0.000, and 0.022 (*p* = .023); For the SNP rs571296, the adjusted BMD values for the three genotypes AA, AG, and GG in the samples studied were as follows: 0.025, 0.005, and −0.012 (*p* = .048).

Four SNPs, rs686708, rs571296, rs6938235, and rs17078405, showed nominal significant associations with hip BMD (*p* < .05; Table 2), but these associations became nonsignificant after multiple-testing correction (significance-threshold *p* values were set at .0074 by the program SNPSpD based on Nyholt's method(23)). All nine SNPs showed nominal significant associations with spine BMD (*p* < .05; Table 2), but after multiple-testing correction (threshold *p* = .0074), only the association of SNPs rs686708 and rs571296 with spine BMD remained significant (*p* = .0010 and *p* = .0068, respectively).

For the SNP rs686708 (A/G), which showed the strongest evidence of association with spine BMD, its allele A was associated with lower spine BMD values in the studied subjects. The subjects with the AA or AG genotypes had significant lower spine BMD values than those with the GG genotype (raw spine BMD values were 1.016, 1.032, and 1.055 g/cm^2^ for the AA, AG, and GG genotypes, *p* = .029), showing an allele dose effect of 0.02 g/cm^2^ lower spine BMD value per copy of the A allele. For the SNP rs571296 (A/G), its minor allele G was associated with lower spine BMD values. Subjects with the GG or AG genotypes had significant lower spine BMD values than those with the AA genotype (raw spine BMD values were 1.061, 1.037, and 1.019 g/cm^2^ for AA, AG, and GG genotypes, *p* = .032), showing an allele dose effect of 0.021 g/cm^2^ lower spine BMD value per copy of the G allele. Linkage disequilibrium (LD) analysis based on the HapMap data set with Haploview software showed the existence of extended and strong intermarker LD in the genomic region between SNP rs571296 and SNP rs339321 (Supplemental Fig. S). SNP rs686708 was outside the LD block and linked very weakly with other SNPs presented (Supplemental Fig. 1).

## Discussion

GPRC6A is a widely distributed member of the amino acid– and calcium-sensing receptor family that appears to function as an nutrient-sensing anabolic receptor.(6) In this study we have demonstrated direct effects of GPRC6A on regulating osteoblast function. The predominant effect of GPRC6A deficiency in vivo was to impair bone mineralization, which was associated with a reduction in osteoblast gene expression markers without changing bone-resorptive markers in vivo. We show that these changes are due, at least in part, to direct effects of the loss of GPRC6A in osteoblasts. In this regard, a primary GPRC6A-dependent effect on osteoblasts is suggested by the abnormal differentiation and attenuated response to calcium and arginine stimulation of osteoblasts derived from *GPRC6A* null mice in vitro. A direct role of GPRC6A in osteoblasts also was confirmed by siRNA-mediated knockdown of this receptor in MC3T3-E1 osteoblasts that was associated with reduction in calcium-stimulated ERK activation. The human relevance of these observations is supported by the association between SNPs rs686708 and rs571296 and reductions in spinal BMD in Caucasians, raising the possibility that *GPRC6A* polymorphisms may contribute to human osteopenia.

Several limitations of this study prevent more definitive conclusions. First, since *GPRC6A* null mice represent a global knockout of this receptor, our in vivo findings are still confounded by the potential actions of concomitant alterations in the ratio of testosterone and estrogens known to be present in these mice or to other undefined secondary consequences of the generalized loss of *GPRC6A.*(6) The absence of increased bone resorption in *GPRC6A* null mice, however, along with the direct in vitro effects of GPRC6 on osteoblast function in two different models (*GPRC6A* null and *GPRC6A* knockdown), suggests that direct effects from receptor loss rather than alterations in sex hormones may account for the osteopenia in *GPRC6A*^−/−^ mice. Indeed, testosterone deficiency typically leads to increased osteoclast-mediated bone resorption,(26) which was not observed in *GPRC6A* null mice. While the attenuation of calcium and amino acid response in isolated osteoblasts from *GPRC6A* null mice supports a direct role, further studies that compare the effects of testosterone replacement with administration of GPRC6A ligands (e.g., calcium, strontium, amino acids, and osteoclacin) to restore bone mass in *GPRC6A*^−/−^ mice will be needed to determine the relative contribution of secondary alterations in sex hormones and primary loss of calcium-sensing receptor responses to the observed bone phenotype in *GPRC6A* null mice. Second, with regard to the role of GPRC6A in humans, SNPs rs686708 and rs571296 are not in the coding region of *GPRC6A* and therefore are not definitely the actual disease-causing variants. Fine mapping of SNPs tightly linked to SNPs rs686708 and rs571296 and functional studies will be required to identify the causal variants in the *GPRC6A* region responsible for human osteopenia.

Our data also support the presence of more than one calcium-sensing mechanism in osteoblasts. Indeed, the full complement of physiologically relevant receptors mediating the changes in amino acids, calcium, and osteocalcin released by osteoclastic-mediated bone resorption continues to be explored. Recent data support a role for the amino acid– and calcium-sensing receptor CASR in regulating osteoblast function.(27,28) Conditional deletion of the 7-TM domain of *CASR* using *Col*1-2.3*-Cre* results in a severe skeletal phenotype.(28) In addition, some studies have implicated CASR in the differentiation of osteoblasts and growth plate chondrocytes.(28,29) On the other hand, the original *CASR* knockout mouse model (which lacks exon 5) fails to display a bone phenotype when hyperparathyroidism is corrected by either performing a “molecular parathyroidectomy” or ablating PTH receptor signaling.(30,31) Also, the anabolic bone effects of strontium ranelate on osteoblast replication and survival are independent of CASR.(32) The explanation for disparity in bone phenotype between the conditional and global *CASR*^−/−^ lacking exon 5 knockout mouse models is not clear. One possibility is that global *CASR*^−/−^ mice lacking exon 5 are hypomorphic owing to the persistent function of an alternatively spliced exon 5–deleted CASR in bone and cartilage. Although *CASR* null osteoblasts retain their calcium-sensing capabilities, so far no signal-transduction activity of the transfected alternatively spliced exon 5–deleted CASR has been identified.(33,34) Another theoretical possibility is that the actions of cre-recombinase in the conditional *CASR* model creates a secreted extracellular domain of CASR that acts as a dominant-negative “decoy” receptor to disrupt CASR function in nearby tissues. Alternatively, the expression of *GPRC6A* in bone and osteoblasts and the resulting bone phenotype raise the possibility that GPRC6A is a candidate for another osteoblastic calcium-sensing receptor(4,15) that is distinct from CASR(15) and which could account for some of these disparate observations. The residual calcium-sensing functions in *GPRC6A* null mice, after silencing of *GPRC6A* in MC3T3 cells, leaves open the possibility of more than one calcium-sensing receptor in osteoblasts.

Nevertheless, the wide expression of *GPRC6A* also raises the possibility that GPRC6A could have a physiologic function in coordinating the responses of bone with other organ systems to changing nutritional cues. For example, there may be a physiologic need to coordinate renal calcium excretion and osteoblast-mediated bone formation. Theoretically, a primary decrease in bone formation and decreased buffering capacity for calcium, with consequent increased urinary expression of dietary calcium, could account for the relationship between osteopenia and hypercalciuria in *GPRC6A*^−/−^ mice. Conversely, activation of GPRC6A would be predicted to stimulate osteoblast-mediated bone calcium accretion and renal calcium conservation to meet the need for bone mineralization. There are some enigmatic clinical disorders with features similar to those of *GPRC6A*^−/−^ mice that support the possibility of coordinated effects between bone formation and renal conservation of calcium. In this regard, a subset of male patients with idiopathic osteoporosis described by Zerwekh and Pak(35) has the combined features of decreased osteoblast-mediated bone formation and hypercalciuria without evidence of hypogonadism, secondary hyperparathyroidism, or abnormal vitamin D levels. Some patients with what appears to be primary hypercalciuria and nephrolithiasis also have concomitant low bone density and low bone turnover(36) without increased PTH levels that would be expected from a sole defect in renal calcium handling. There are also examples of primary bone defects that are associated with hypercalciuria without increased PTH. These include osteogenesis imperfecta type 1 caused by *Col*1A1 and *Col*1A2 mutations, infantile hypophoshatasia caused by inactivating mutations of *ALP*, and McCune-Albright syndrome caused by activating mutations of *GNAS1.*(8) It will be interesting to determine if gene polymorphisms of *GPRC6A* are associated with osteopenic and hypercalciuric clinical disorders.

These findings also expand the repertoire of GPCRs that regulate the functional activity and provides greater insights into the function of Gαi in bone. In this regard, GPRC6A is predominately a Gαi-coupled receptor.(4) Compared with other well-characterized anabolic receptors, such as the PTH receptor, which is coupled to Gαs and Gαq-dependent signaling pathways(37) that regulate proliferation and differentiation of osteoblasts, these studies suggest that Gαi-dependent pathways play a distinct role in regulating osteoblast gene expression, mainly affecting the mineralization of bone. The classica effect of Gαi activation in inhibiting adenylyl cyclase activity might be expected to oppose signaling of Gαs-coupled GPCRs in osteoblasts.(38) There is little available information on the role of osteoblast signaling by Gαi, but the pertussis toxin–dependent proliferative actions of fluoride and strontium on osteoblasts in vitro,(4,39) Gαi-coupled apelin receptor stimulation of osteoblast proliferation in vitro.(40) development of osteoporosis in Gαi-coupled CB2 cannabinoid receptor knockout mice,(41) as well as our findings in *GPRC6A* null mice, suggest that Gαi signaling is important. Potential anabolic actions of Gαi-coupled receptors, including GPRC6A, might be mediated through activation of mitogen-activated protein kinase (MAPK) pathways.(42)

In contrast to our studies, Wellendroph and colleagues(16) generated a *GPRC6A* null mouse by targeting exon VI of *GPRC6A*, which encodes the seven transmembrane domain and C-terminal tail. Analysis of these mice failed to identify any skeletal abnormalities in 13-week-old mice. At present, we have no explanation for these disparate findings, but similar to the differences observed between existing *CASR* knockout models described earlier,(28,43) this may represent another example where disrupting the extracellular domain results in no expression of the GPCR, whereas disrupting the transmembrane domain may lead to partial translation of the extracellular domain and functional effects. Regardless, there are other instances of multiple laboratories independently targeting the same GPCR and failing to find similar phenotypes that have been attributed to a variety of different mechanisms.(44)

In summary, we have shown that GPRC6A has a direct function in osteoblasts. The ligand profile of GPRC6A, which includes extracellular calcium, calcimimetics, amino acids, and osteocalcin,(1,2,4,45) along with the complex phenotype of *GPRC6A* null mice, suggests that GPRC6A may represent an anabolic receptor that responds to a variety of nutritional and hormonal signals and may serve to coordinate the functions of multiple organs, including bone, to changes in the local and systemic concentrations of these ligands.
